# Biogeographic Patterns and Assembly Mechanisms of Bacterial Communities Differ Between Habitat Generalists and Specialists Across Elevational Gradients

**DOI:** 10.3389/fmicb.2019.00169

**Published:** 2019-02-11

**Authors:** Zhengming Luo, Jinxian Liu, Pengyu Zhao, Tong Jia, Cui Li, Baofeng Chai

**Affiliations:** ^1^Institute of Loess Plateau, Shanxi University, Taiyuan, China; ^2^Department of Geography, Xinzhou Teachers University, Xinzhou, China; ^3^Department of Environment and Economics, Shanxi University of Finance and Economics, Taiyuan, China

**Keywords:** community assembly, niche breadth, elevational gradient, specialists and generalists, stochastic/deterministic processes

## Abstract

A core issue in microbial ecology is the need to elucidate the ecological processes and underlying mechanisms involved in microbial community assembly. However, the extent to which these mechanisms differ in importance based on traits of taxa with different niche breadth is poorly understood. Here, we used high-throughput sequencing to examine the relative importance of environmental selection and stochastic processes in shaping soil bacterial sub-communities with different niche breadth (including habitat generalists, specialists and other taxa) across elevational gradients on the subalpine slope of Mount Wutai, Northern China. Our findings suggested that the composition of soil bacterial communities differed significantly different among elevational gradients. According to the niche breadth index, 10.9% of OTUs were defined as habitat generalists (B-value >8.7) and 10.0% of OTUs were defined as habitat specialists (B-value <1.5). Generalists and specialists differed distinctly in diversity and biogeographic patterns across elevational gradients. Environmental selection (deterministic processes) and spatial factors (stochastic processes) seemed to determine the assembly and biogeography of habitat generalists. However, for specialists, deterministic processes strongly influenced the distribution, while stochastic processes were not at play. Environmental drivers for generalists and specialists differed, as did their importance. Elevation, total nitrogen and pH were the main factors determining habitat generalists, and soil water content, nitrate nitrogen and pH had the strongest impacts on specialists. Moreover, variation partitioning analysis revealed that environmental selection had a much greater impact on both generalists (17.7% of pure variance was explained) and specialists (3.6%) than spatial factors. However, generalists had a much stronger response to spatial factors (2.3%) than specialists (0.3%). More importantly, null models of β-diversity suggested that specialists deviated significantly from non-neutral assembly mechanisms (relative null deviation= 0.64–0.74) relative to generalists (0.16–0.65) (*P* < 0.05). These results indicate that generalists and specialists are governed by different assembly mechanisms and present distinct biogeographical patterns. The large proportion of unexplained variation in specialists (93.3%) implies that very complex assembly mechanisms exist in the assembly of specialists across elevational gradients on the subalpine slope of Mount Wutai. It is essential to understand the microbial community assembly at a more refined level, and to expand the current understanding of microbial ecological mechanisms.

## Introduction

Understanding the mechanisms underlying the community assembly, which shapes the complicated biogeographical patterns of microbes, is a continuing topic of debate in microbial ecology (Nemergut et al., 2013; Zhou and Ning, 2017). The niche-based theory has always assumed that deterministic abiotic and biotic factors as environmental conditions, biotic interaction (e.g., predation, competition, and symbiosis), habitat heterogeneity and species traits (e.g., abundance, metabolism and morphology) determine the composition of community, and these are often collectively referred to as deterministic processes (Zhou and Ning, 2017). In contrast, neutral theory hypothesizes that community structures are independent of species traits and governed by stochastic processes (e.g., birth, death, speciation, extinction, colonization and dispersal limitations) (Vázquez et al., 2002; Chave, 2010; Zhou and Ning, 2017). Many microbial ecology studies have shown that deterministic and stochastic processes work together simultaneously, and are co-responsible for microbial community assemblies (Caruso et al., 2011; Maren et al., 2017; Zhou et al., 2017). However, there are still open questions regarding how to properly uncover the biogeography of microbial communities, as well as how to elucidate the importance of deterministic and stochastic processes for community assembly (Liao et al., 2016), not only qualitatively (Vanwonterghem et al., 2014), but also quantitatively (Stegen et al., 2013).

Many studies of microbial community assembly mechanisms have tended to associate one single model with the entire metacommunity without making any systematic distinctions among different categories of species (Ferrenberg et al., 2013; Dini-Andreote et al., 2015; Valyi et al., 2016; Liu et al., 2018). However, some species that are often referred to as habitat generalists exhibit broad environmental tolerances, while others that are defined as habitat specialists have different community traits, exhibiting very specific and narrow environmental tolerances (Pandit et al., 2009; Liao et al., 2016). It has been reported that different mechanisms occur simultaneously during the bacterial community assembly (Caruso et al., 2011; Langenheder and Székely, 2011; Székely and Langenheder, 2014; Maren et al., 2017). Therefore, it is possible that different properties or traits of bacterial groups may assemble via different mechanisms. Pandit et al. (2009) found that for zooplankton communities, to a larger degree, generalists were assembled by dispersal-related mechanisms, while, specialists were mostly assembled by deterministic processes. Kneitel (2018) assessed niche-based predictions of occupancy and environmental responses using invertebrates from California vernal pools and found that they depended on dispersal traits for both generalists and specialists.

Several studies have compared the relative importance of deterministic and stochastic processes for assembly of habitat generalists and specialists in microbial communities in different ecosystems (Székely and Langenheder, 2014; Liao et al., 2016; Monard et al., 2016). For both habitat generalists and specialists, some studies have revealed that deterministic processes comprised the most important assembly mechanism, whereas dispersal processes did not (Székely and Langenheder, 2014). In contrast, others have argued that the assembly of habitat specialists were governed mainly by environmental processes, while habitat generalists were predominantly structured by dispersal processes (Pandit et al., 2009). By analyzing the dominant habitat specialists and generalists within the three domains of life (fungi, bacteria, and archaea) across a terrestrial-freshwater gradient, Monard et al. (2016) identified deterministic processes as a dominant assembly mechanism for specialists within all three domains and showed that their dispersal was limited. It has been suggested that habitat specialists and generalists have different ecological responses to environmental changes in multiple ecosystems (Stilmant et al., 2008; Székely and Langenheder, 2014; Liao et al., 2016; Monard et al., 2016; Kneitel, 2018). Therefore, elucidating and distinguishing the assembly mechanisms of bacterial subcommunities based on organism distribution patterns (niche breadth) could further contribute to the understanding of the bacterial community assembly and shaping of bacterial biogeography.

The elevation gradient influences various environmental factors such as temperature, moisture and light, and its impact on environmental factors is 1,000 times greater than that of the latitude gradient, which is of great significance for biodiversity conservation and investigations of biodiversity distribution patterns and their driving factors (Körner, 2007). Elevational gradients are characterized by distinct climate and ecological changes over short geographic distances and therefore offer a unique platform to improve the understanding of basic processes relevant to community assembly (Ren et al., 2018). Mount Wutai Natural Reserve, located in northeastern Shanxi Province, is one of the best preserved natural ecosystems and the highest peak in North China. Topographic and climatic variations result in a distinct vertical zonation of major forest types in Mount Wutai, especially along the northern slope (Liu et al., 2003; Dai et al., 2005). These characteristics make Mount Wutai an ideal area to study the microbial biogeography and the mechanism of bacterial community assembly. The elevational patterns of soil microbial communities have recently attracted a great deal of interest (Yao et al., 2017a; Zhang et al., 2018); however, the elevational distribution patterns and mechanisms of soil microbial subcommunities with different niche breadth traits across elevational gradients are still poorly understood. Therefore, this study was conducted to : (1) describe the diversity, structure and biogeographical patterns of soil bacterial communities and subcommunities (specialists, generalists and other taxa) across elevational gradients along the slope of Mount Wutai; (2) explore the variations and drivers related to the community assembly of soil bacterial subcommunities among elevational gradients; (3) quantify the relative roles of environmental selection and space distance in the community assembly of soil bacterial taxa and differentiate the community assembly mechanisms of bacterial subcommunities with different niche breadth across elevational gradients.

## Materials and Methods

### Study Sites and Soil Sampling

**Figure 1:**
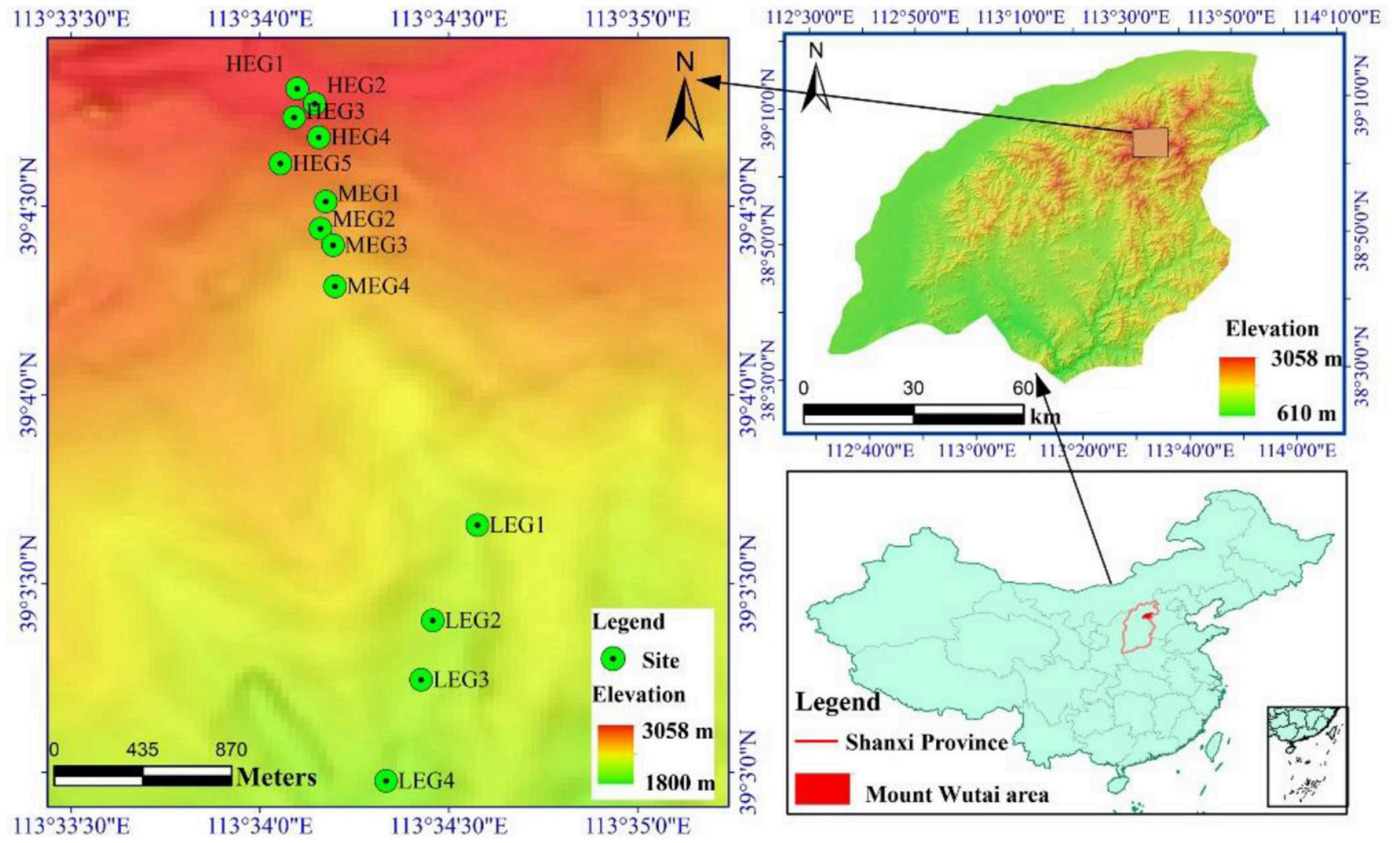
Map showing the location of Mount Wutai and the sampling sites. High elevational gradient (HEG) (2900–3055 m) including five different elevation sampling sites (HEG1, HEG2, HEG3, HEG4, and HEG5), both medium elevational gradient (MEG) (2500–2800 m) (MEG1, MEG2, MEG3, and MEG4) and low elevational gradients (LEG) (2000–2300 m) (LEG1, LEG2, LEG3, and LEG4) including four different elevation sampling sites.

Mount Wutai (112°48′-113°55′E; 38°27′-39°15′N) is located in the northeast Shanxi Province, Northern China (Figure 1). The climate is warm temperate semi-humid. As the elevational gradient increases from 934 to 3058 m a.s.l, the mean annual precipitation increases from 391 to 840 mm, and the mean annual temperature decreases from 7.2°C to −4.9°C (Dai et al., 2005). Mount Wutai also reaches the climatically controlled alpine timberline in the temperate broadleaved forest zone in China (Liu et al., 2003; Dai et al., 2005). There are five distinct vegetation types along the elevational gradient on the northern slope of Mount Wutai; namely; frigid subalpine and alpine meadows (above 2800 m a.s.l), subfrigid subalpine scrub meadows (2500–2800 m a.s.l), cold temperate coniferous forests (1800–2500 m a.s.l), temperate coniferous and broad-leaved mixed forests (1400–1800 m a.s.l) and warm-temperate scrub-grasslands (900–1400 m a.s.l).

Soil samples were collected from the subalpine slope of Wutai Mountain on August 2017 at 13 elevations (2085, 2172, 2221, 2295, 2581, 2657, 2707, 2728, 2900, 2950, 3019, 3031, and 3051m) (Table S1), representing three elevational gradients (Table S1); namely, high elevational gradient (HEG) (2900–3055 m a.s.l), medium elevational gradient (MEG) (2500–2800 m a.s.l) and low elevational gradient (LEG) (2000–2300 m a.s.l) (Table 1).

**Table 1:** Description of soil physicochemical parameters among three elevational gradients.

**Parameters**	**HGE**	**MGE**	**LGE**
pH	5.85 ± 0.04b	5.86 ± 0.07b	6.32 ± 0.14a
ST (°C)	12.39 ± 0.23c	15.02 ± 0.23b	18.12 ± 0.46a
SWC (%)	37.53 ± 1.86a	42.61 ± 2.07a	25.57 ± 3.52b
EC (us/cm)	130.27 ± 10.22a	97.50 ± 5.76b	96.92 ± 10.33b
TN (%)	0.52 ± 0.02b	0.63 ± 0.05a	0.45 ± 0.03b
TC (%)	5.65 ± 0.22b	8.07 ± 0.41a	6.01 ± 0.27b
C/N ratio	10.86 ± 0.17b	13.41 ± 0.98a	13.62 ± 0.50a
SOC (g/kg)	41.68 ± 0.98a	38.95 ± 1.31a	38.51 ± 1.50a
${\text{NH}}_{4}^{+}$-N (g/kg)	48.1 ± 2.72b	70.99 ± 2.40a	49.8 ± 3.04b
${\text{NO}}_{3}^{-}$-N (g/kg)	4.40 ± 0.16c	5.27 ± 0.42b	6.56 ± 0.40a
${\text{NO}}_{2}^{-}$-N (g/kg)	1.48 ± 0.04a	1.47 ± 0.07a	1.48 ± 0.10a

*Values are the means (±Standard error). Different letters represent significant differences in P < 0.05 level between different samples. HEG, High elevational gradient; MEG medium elevational gradient; LEG, low elevational gradients*.

At each elevation, soil samples of the topsoil (0–10 cm) were collected from three plots (1 m × 1 m) as three independent replicates. Five subsamples (one taken at each corner and one at the center) were collected from each plot, then mixed into a single sample in a polyethylene bag. Most roots, animals and stones were removed by 2 mm mesh screen, after which samples were divided in two. One part was then air-dried for physiochemical analysis, while the other part was preserved at −80°C for molecular analysis. In each plot, plant species composition included the species richness and the number of each plant species was measured.

### Soil Physical and Chemical Analyses

Soil water content (SWC) was measured gravimetrically by oven drying to constant mass at 105°C. Electrical conductivity (EC) and soil temperature (ST) at 10 cm depth were measured with a portable soil parameter detector (HA-TR-III, China). Soil total carbon (TC) and total nitrogen (TN) were quantified by elemental analysis (Elementar Vario MACRO, Germany); Soil organic carbon (SOC) were measured using the K_2_Cr_2_O_7_ oxidation method (Walkley, 1947). Soil pH was quantified in 1 M KCl soil suspension [soil: water ratio of 1:2.5(w/v)] (HANNA, Italy). Ammonium nitrogen (${\text{NH}}_{4}^{+}$-N), nitrate nitrogen (${\text{NO}}_{3}^{-}$-N), nitrite nitrogen (${\text{NO}}_{2}^{-}$-N) were quantified by automated discrete analysis (CleverChem 380, Germany).

### DNA Extraction, PCR Amplification, and Miseq Sequencing

We used 0.5 g amounts of each soil sample to extract DNA using the E.Z.N.A.® Soil DNA Kit (Omega Bio-tek, Norcross, GA, USA) following the manufacturer's instructions and were quantified using NanoDrop ND-1000 UV-Vis Spectrophotometer (NanoDrop Technologies, Wilmington, DE, USA). A total of thirteen samples (mixing the three repeats for a sample in each site) were analyzed. The primer set 338F (5′-ACTCCTACGGGAGGCAGCA-3′) and 806R (5′-GGACTACHVGGGTWTCTAAT-3′) was used to amplify the V3-V4 hyper variable region of 16S rRNA gene in bacteria. The specific conditions of PCR amplification and purification can be referred to the article published by our research group (Liu et al., 2018). After purification and quantification, a mixture of amplicons was sequenced on an Illumina Miseq sequencer according to standard protocol. The sequencing and bioinformatics service were performed by Majorbio Bio-pharm Technology Co., Ltd., Shanghai, China.

### Bioinformatics Analysis

The obtained raw sequence data were processed using the Quantitative Insights into Microbial Ecology (QIIME) pipeline, the procedures were described in detail by Yao et al. (2017b). A total of 828886 sequences were obtained for the 13 samples. Operational taxonomic units (OTUs) were picked 158 at the 97% identity level using CD-HIT in the QIIME pipeline (Li and Godzik, 2006). The bacterial OTUs were taxonomically identified using the Silva128 16S rRNA database. Random resampling was performed at a depth of 22310 sequences per sample. The bacterial sequences have been deposited in the SRA of the NCBI database under the Accession No. SRP135838.

### Calculation of Niche Breadth

The niche breadth was calculated as described by Pandit et al. (2009) using Levins' niche breadth index (Levins, 1968):

(1) $$\begin{array}{l} {\text{B}}_{j}=1/\overset{n}{\underset{i=1}{\sum }}P_{ij}^{2} \end{array}$$

where B_*j*_ indicates the habitat niche breadth of OTU_*j*_ and *P*_*ij*_ is the relative abundance of OTU_*j*_ in a given habitat i (i.e., one of the 13 sampling sites). The average B-values were calculated from the entire soil bacterial community as an index of habitat niche breadth at the community level. OTUs with mean relative abundances <2 × 10^−5^ were removed, as they could erroneously indicate specialists (Pandit et al., 2009). OTUs with a B-value >8.7 were considered as habitat generalists that were present and more evenly distributed along a wider range of habitats, while OTUs with a B-value <1.5 were defined as habitat specialists (Logares et al., 2013). B-values >8.7 and B-value <1.5 were chosen because both values were within the outlier area of the B distribution (Figure S1).

INDVAL analysis was performed using the labdsv package within the R program (https://www.r-project.org) to further select strict habitat specialists (Dufrene and Legendre, 1997). OTUs with significant (*P* < 0.05) INDVAL values >0.3 among the specialists determined by niche breadth were considered strict specialists and included in subsequent analyses (Liao et al., 2016).

### Statistical Analysis

The Chao1 estimator, observed OTUs, Shannon index and Simpson index were calculated using the PAST software (v3.10). One-way analysis of variance (ANOVA) was conducted to assess the differences in the environmental parameters, alpha diversity indices and the relative abundance of dominant bacterial phyla among the three elevational gradients. Principal co-ordinates analysis (PCoA) was used to analyze differences in bacterial community structure. Analysis of similarity (ANOSIM) was performed to examine the significant differences in soil bacterial communities among the three elevational gradients [Vegan package in R (Oksanen et al., 2013)]. Redundancy analysis (RDA) or canonical correspondence analysis (CCA) was also used to identify the correlations among environment variables and the bacterial community composition. Before RDA or CCA, forward selection of the environmental variables was performed using stepwise regression and the Monte Carlo Permutation Test (CANOCO for Windows Version 5.0). The environmental variables that were statistically significant (*P*< 0.05) were selected based on forward selection. The principal coordinates of neighbor matrices (PCNM) were calculated to reflect the spatial distance, and the most significant PCNM variables were chosen by conducting forward selection procedures using the PCNM function in the R vegan package (Oksanen et al., 2013). The Mantel and partial Mantel test were used to assess the correlations among bacterial communities with environmental variables and spatial distances.

The contribution of environmental factors and space distance with the variations in the four groups (entire bacterial community and three subcommunities) were measured by variance partitioning analysis (VPA) (CANOCO for Windows Version 5.0). The variables from each part (i.e., environmental and spatial variables) were forward selected before VPA (Mcardle and Anderson, 2001).

The β-diversity null deviation approach uses a null model to create stochastically assembled communities from the regional species pool to determine the degree to which the observed β-diversity patterns deviate from stochastic assembly (Chase and Myers, 2011; Tucker et al., 2016). Hence, this approach can assess changes in β-diversity that result from the relative influences of deterministic and stochastic processes. We measured the null deviation as the relative difference of the observed β-diversity from the null-model β-diversity. For each sample, the expected β-diversity under the null model was calculated from 999 stochastically assembled communities in the “reldist,” “vegan” and “bipartite” R package.

## Results

### Soil Characteristics Among Elevational Gradients

The patterns of the soil properties co-varied with the vegetation types (Table 1). Soil temperature (ST) and ${\text{NO}}_{3}^{-}$-N varied significantly among three elevational gradients (*P* < 0.05) (Table 1). The soil pH, ST, C/N ratio, and ${\text{NO}}_{3}^{-}$-N in low elevational gradient (LEG) had the highest values (pH = 6.32, ST = 18.12°C, C/N ratio = 13.62 and ${\text{NO}}_{3}^{-}$-N = 6.56 g·kg^−1^), and the high elevational gradient (HEG) had the lowest values (pH = 5.85, ST = 12.39°C, C/N ratio = 10.86 and ${\text{NO}}_{3}^{-}$-N = 4.40 g·kg^−1^). EC exhibited the lowest values (97.5 us/cm) in LEG soils (*P* < 0.05). TN (0.63%), ${\text{NH}}_{4}^{+}$-N (7.1%) and TC (8.07%) in medium elevational gradient (MEG) soils were significantly higher than in HEG and LEG soils (*P* < 0.05). SWC in MEG (42.61%) was highest, and was significantly higher than in LEG (25.57%) soils (*P* < 0.05) (Table 1). There were no significant differences in SOC and ${\text{NO}}_{2}^{-}$-N among three elevational gradients (all *P* > 0.05) (Table 1).

### Composition and Taxonomy of Bacterial Communities

**Figure 2:**
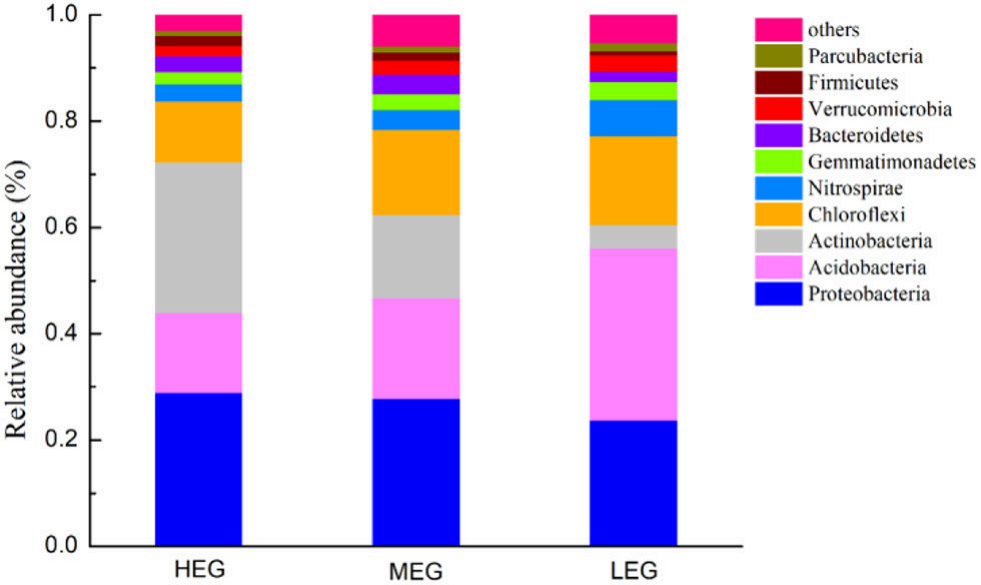
Relative abundance of the dominant bacterial phyla (with average relative abundance > 1%) among three elevational gradients. HEG high elevational gradient, MEG medium elevational gradient, LEG low elevational gradients.

A total of 3764 bacterial OTUs were identified from 828,886 high-quality sequences in soil samples. Forty-five phyla were identified, with 13 dominant phyla of relative abundances >1%. The dominant bacterial phyla in all samples were *Proteobacteria, Acidobacteria, Actinobacteria, Chloroflexi, Nitrospirae, Gemmatimonadetes, Bacteroidetes, Verrucomicrobia, Firmicutes*, and *Parcubacteria* (relative abundance > 1%, Figure 2) and these dominant phyla occupied more than 95% of the bacterial sequences in each of the soils of three elevational gradients. The relative abundances of the dominant bacterial phyla varied among the three elevational gradients (Figure 2). *Acidobacteria, Actinobacteria, Nitrospirae, Bacteroidetes*, and *Firmicutes* exhibited significantly different relative abundances among elevational gradients (all *P* < 0.05) (Figure S2). Additionally, the relative abundances of *Proteobacteria, Actinobacteria*, and *Firmicutes* increased with elevation, while those of *Acidobacteria, Chloroflexi, Nitrospirae, Gemmatimonadetes, Verrucomicrobia*, and *Parcubacteria* decreased with elevation.

**Figure 3:**
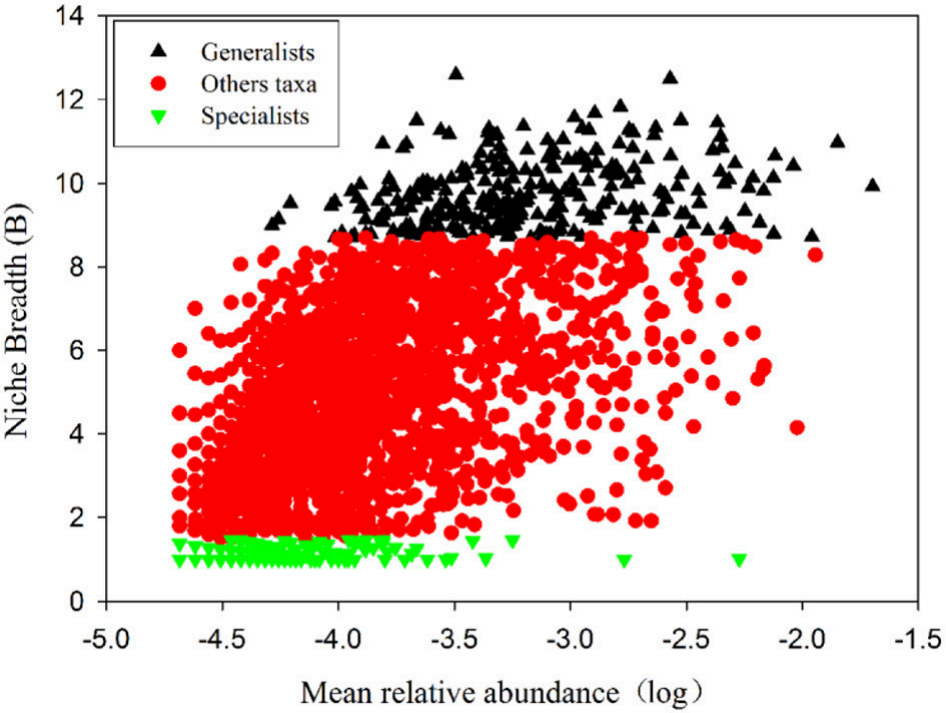
Habitat specialists and generalists on the northern slope of Mount Wutai. Each dot represents an OTU. The *x* axis indicates mean relative abundances and the *y* axis indicates Niche Breadth (B). Generalists (black; B> 8.7), Specialists (green; B < 1.5) and Other taxa (red;1.5 ≤ B ≤8.7) OTUs are indicated.

**Figure 4:**
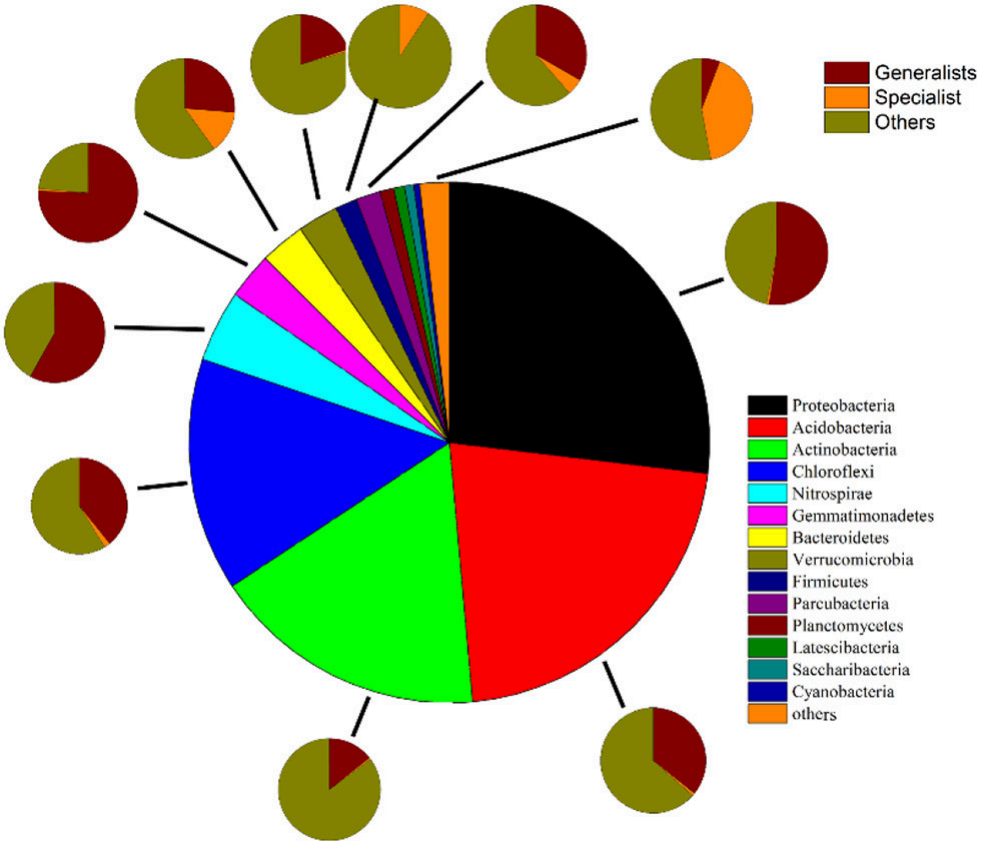
Phylum compositions of all taxa, specialists, generalists and other taxa. The largest pie in the middle shows the phylum compositions of total bacteria. Sequences that are not classified or can be classified into known groups but have a mean relative abundance <0.1% are assigned to “others.” The eleven smaller pies show the proportions of specialists, generalists and the remaining groups belonging to *Proteobacteria, Acidobacteria, Actinobacteria, Chloroflexi, Nitrospirae, Gemmatimonadete, Bacteroidetes, Verrucomicrobia, Parcubacteria, Firmicutes*, and others.

Removing OTUs with mean relative abundances <2 × 10^−5^ left 2808 OTUs to be classified based on niche breadth. Among these, 307 specialist OTUs (10.9%), 280 generalist OTUs (10.0%) and 2221 other taxa OTUs (79.1%) were detected (Figure 3 and Table S4). In all taxa, *Proteobacteria* (27.01%) was detected as the most abundant phylum, followed by *Acidobacteria* (21.74%), *Actinobacteria* (17.19%), *Chloroflexi* (14.53%) and *Nitrospirae* (4.46%) (Table S4). A much higher proportion of *Proteobacteria* (pi of generalists 14.14% vs. specialists 0.22%, p_i_ represents the mean relative abundance of i phylum), *Acidobacteria* (pi of generalists 7.77% vs. specialists 0.18%), *Actinobacteria* (pi of generalists 2.46% vs. specialists 0.09%), *Chloroflexi* (pi of generalists 5.69% vs. specialists 0.29%), *Nitrospirae* (pi of generalists 2.59% vs. specialists 0.005%) and *Gemmatimonadetes* (pi of generalists 2.20% vs. specialists 0.02%) was presented within generalists than specialists (Figure 4, Table S4).

According to the observed and estimated OTUs (Chao1), HEG was inhabited by the richest bacterial communities, while LEG had the least bacterial richness for all taxa, generalists and other taxa (all *P* < 0.05; Table 2). In contrast, the OTUs richness and Chao1 of specialists were highest in LEG, followed by MEG, with the lowest value recorded in HEG (*P* < 0.05; Table 2). The Shannon-Wiener indices of all taxa, generalists and other taxa in HEG were significantly greater than those in LEG (*P* < 0.05). The Simpson indices of all taxa and specialists were not significantly different among the three elevational gradients (all *P*>0.05; Table 2). The three alpha diversity indices other than the Simpson index differed among the three elevational gradients (Table 2).

**Table 2:** Phylotype richness and diversity estimators of the microbial communities among three elevational gradients.

**Groups**	**Elevation gradients**	**OTUs**	**Chao-1**	**Simpson**	**Shannon**
All	HGE	1781.40 ± 28.18a	2156.80 ± 40.02a	0.997 ± 0.000a	6.42 ± 0.02a
	MGE	1608.00 ± 67.28b	1926.25 ± 86.49b	0.994 ± 0.002a	6.16 ± 0.14b
	LGE	1438.75 ± 23.58c	1654.00 ± 21.61c	0.993 ± 0.000a	6.02 ± 0.03b
Generalists	HGE	276.60 ± 0.87a	279.56 ± 1.69a	0.987 ± 0.002a	4.90 ± 0.02a
	MGE	274.25 ± 2.32a	276.65 ± 1.80ab	0.983 ± 0.004a	4.77 ± 0.08a
	LGE	264.25 ± 1.31b	272.33 ± 2.31b	0.977 ± 0.005b	4.58 ± 0.04b
Specialists	HGE	15.40 ± 0.68c	39.67 ± 4.99c	0.857 ± 0.027a	2.35 ± 0.12b
	MGE	40.00 ± 12.14b	52.06 ± 13.36b	0.859 ± 0.038a	2.65 ± 0.09b
	LGE	64.25 ± 6.88a	100.91 ± 18.66a	0.949 ± 0.015a	2.65 ± 0.22a
Other taxa	HGE	1489.40 ± 28.23a	1851.00 ± 41.47a	0.996 ± 0.000a	6.26 ± 0.03a
	MGE	1293.75 ± 73.96b	1608.00 ± 92.56b	0.991 ± 0.003ab	5.97 ± 0.15b
	LGE	1110.25 ± 25.05*c*	1305.00 ± 26.32*c*	0.990 ± 0.000b	5.70 ± 0.01c

*Values are the means (± Standard error). Different letters represent significant differences in P < 0.05 level between different elevational gradients. HEG, High elevational gradient; MEG, medium elevational gradient; LEG, low elevational gradients*.

**Figure 5:**
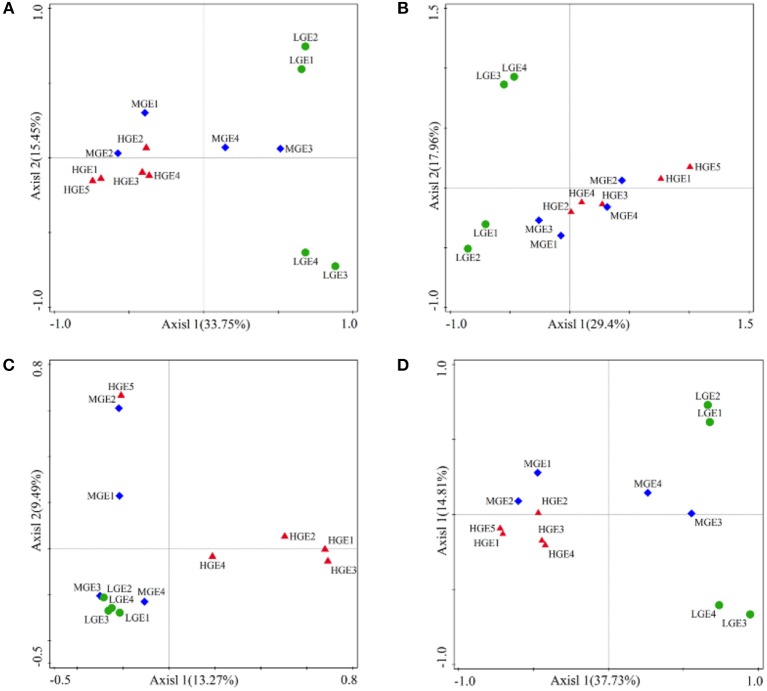
PCoA based on Bray-Curtis similarities of bacterial communities among three elevational gradients. **(A)** All; **(B)** Generalists; **(C)** Specialists; **(D)** Other taxa. High elevational gradient (HEG) (2900–3055 m) including five different elevation sampling sites (HEG1, HEG2, HEG3, HEG4, and HEG5), both medium elevational gradient (MEG) (2500–2800 m) (MEG1, MEG2, MEG3, and MEG4) and low elevational gradients (LEG) (2000–2300 m) (LEG1, LEG2, LEG3, and LEG4) including four different elevation sampling sites.

Variations in the community structure of the four classified bacterial groups among elevational gradients were statistically analyzed using PCoA based on Bray-Curtis (Figure 5). ANOSIM verification indicated the overall community compositions of the four groups were significantly (all *P* < 0.05) separated across the three elevational gradients (Table S3). Pair-wise ANOSIM analysis revealed significant (*P* < 0.05) separation of the bacterial community composition between any of the two elevational gradients except for MEG and LEG (*P*>0.05) with respect to all taxa, specialists and other taxa (Table S3). There were no significant variations in the structure of generalists between HEG and MEG (*P* > 0.05), but obvious variations between MEG and LEG or HEG and LEG (all *P* < 0.05, Table S3).

### Correlations of Soil Bacterial Communities With Environmental and Spatial Factors

**Figure 6:**
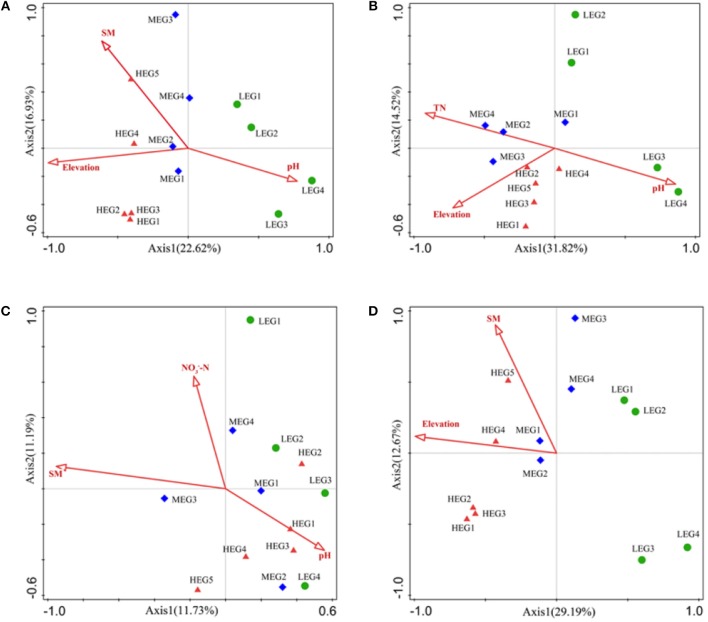
RDA or CCA of the soil microbial community structure and environmental variables. **(A)** All; **(B)** Generalists; **(C)** Specialists; **(D)** Other taxa. Only the environmental variables which were significantly correlated with RDA1, or CCA1 were shown in figures. High elevational gradient (HEG) (2900–3055 m) including five different elevation sampling sites (HEG1, HEG2, HEG3, HEG4, and HEG5), both medium elevational gradient (MEG) (2500–2800 m) (MEG1, MEG2, MEG3, and MEG4) and low elevational gradients (LEG) (2000–2300 m) (LEG1, LEG2, LEG3, and LEG4) including four different elevation sampling sites.

To explore the key environmental drivers shaping soil bacterial communities, environmental variables were analyzed by CCA or RDA. Among the environmental variables, elevation, SWC and pH were identified by RDA as the significant predictors for entire bacterial communities (*P* < 0.05) (Figure 6). The significant environmental variables for specialists differed from those for generalists. ${\text{NO}}_{3}^{-}$-N, SWC and pH were significant predictors for specialists. However, the composition of generalists was significantly influenced by elevation, pH and TN (Figure 6). The results showed that four variables (elevation, SWC, pH, and PCNM1) selected for all taxa, while three variables (elevation, SWC and PCNM1) selected for other taxa, four variables (elevation, pH, TN, and PCNM1) selected for generalists and four variables (${\text{NO}}_{3}^{-}$-N, SWC, pH, and PCNM2) selected for specialists.

**Figure 7:**
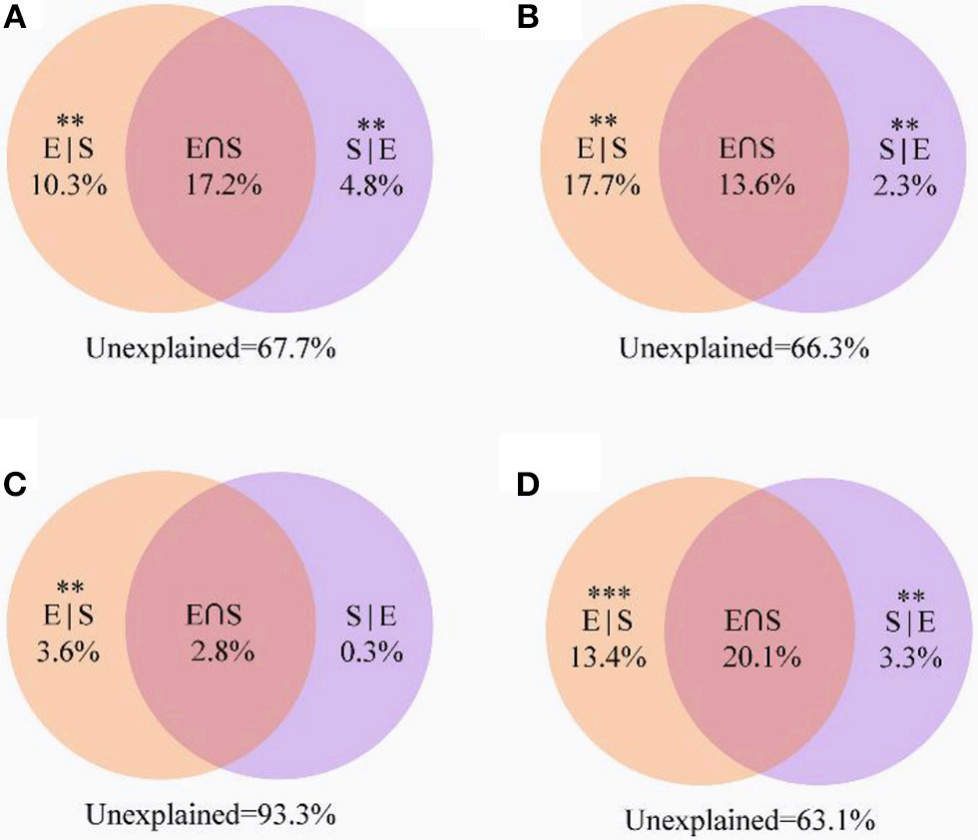
Variation partitioning analysis showing the percentages of variance in soil bacterial communities explained by environmental factors and spatial distance. **(A)** All; **(B)** Generalists; **(C)** Specialists; **(D)** Other taxa. The variation explained by pure spatial and environmental factors corresponds to the bacterial community without the effect of the other by ANOVA permutation tests. ^**^P < 0.01 and ^***^P < 0.001. S|E pure spatial variation, E|S pure environmental variation, S⋂E share explained variation, 1-S|E- E|S-S⋂E unexplained variation.

VPA revealed that 32.3% of the variation for the entire community was significantly explained by environmental and spatial variables. Among them, environmental variables and spatial distance independently explained 10.3 and 4.8%, respectively (Figure 7A). For other taxa, the combination of these variables explained 36.9% of the observed variation (Figure 7D), while environmental variables and spatial distance explained 13.4 and 3.3%, respectively. VPA showed that the explained proportion of purely environmental variation (17.7%) in the composition of generalists tended to be higher than that of purely spatial variables (2.3%). Notably, shared environmental and spatial variables explained 13.6% of the variation in generalists, whereas they only explained 2.8% of the community variation of specialists. More importantly, a large amount of the variation (93.3%) in specialists was not explained by the spatial and environmental variables, and the contribution of purely environmental variables (3.5%) was much more than that of purely spatial variables (0.3%).

**Figure 8:**
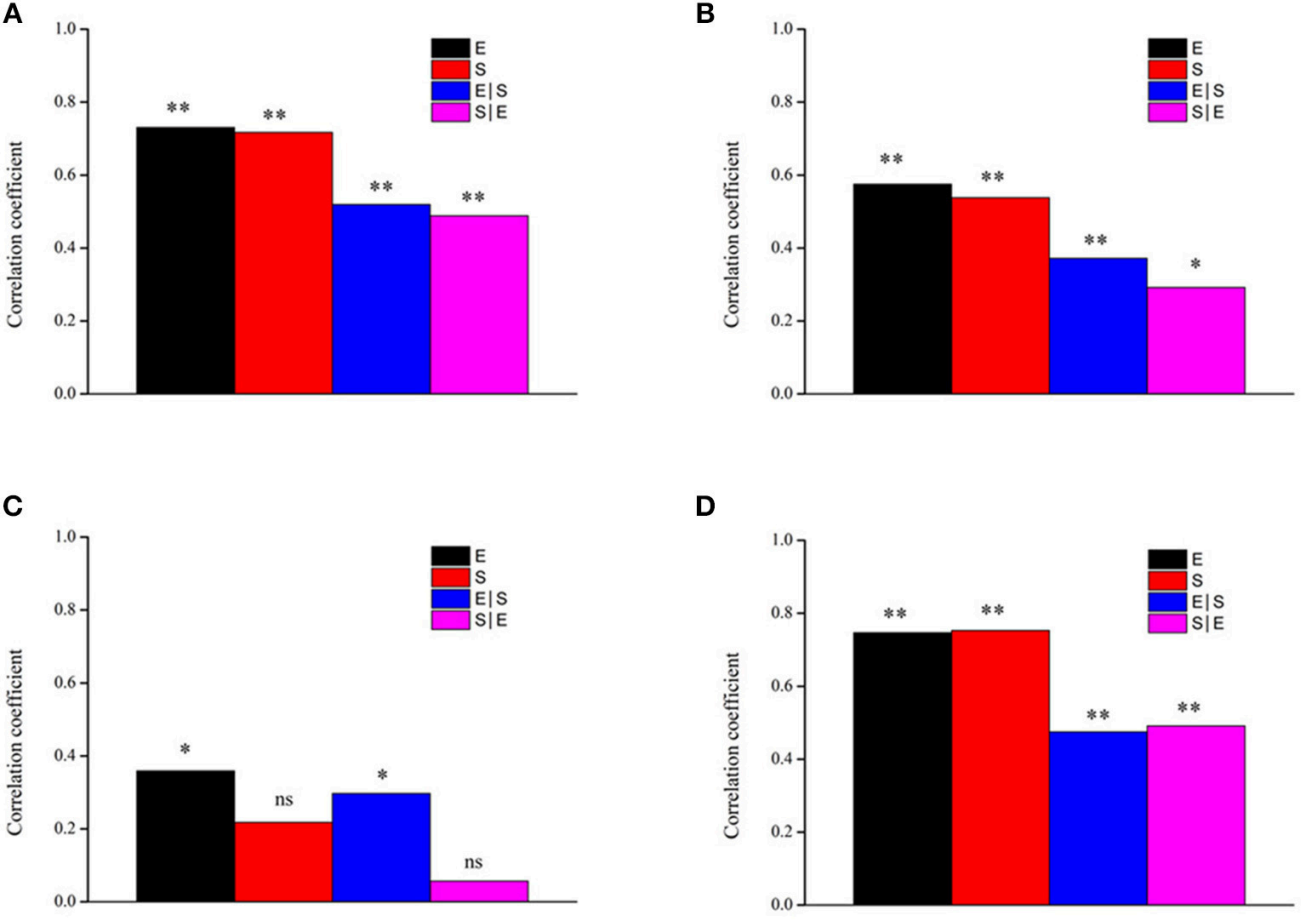
Mantel and partial Mantel tests for the correlation between bacterial community dissimilarity and environmental and spatial factors using Pearson's coefficient. ^*^P < 0.05, ^**^P < 0.01. **(A)** All; **(B)** Generalists; **(C)** Specialists; **(D)** Other taxa. E environmental variables, S spatial variation, S|E pure spatial variation, E|S pure environmental variation, ns not significant.

We further confirmed the effects of environmental variables and spatial distance on the four groups by using the Mantel and partial Mantel test. The results revealed that, when spatial distance was controlled, the structures of the four groups were significantly correlated with environmental variables (all *P* < 0.05; Figure 8). The results also showed significant effects of spatial distances on all taxa, other taxa and generalists (all *P* < 0.05; Figures 8A–D) when the effects of environmental variables were controlled, but the effects of spatial distance on specialists were not significant (*P* > 0.05; Figure 8C). Moreover, both environmental variables and spatial distance were significantly correlated with generalists (*P* < 0.05; Figure 8B), but only environmental variables were positively correlated with specialists (*P* < 0.05; Figure 8C).

**Figure 9:**
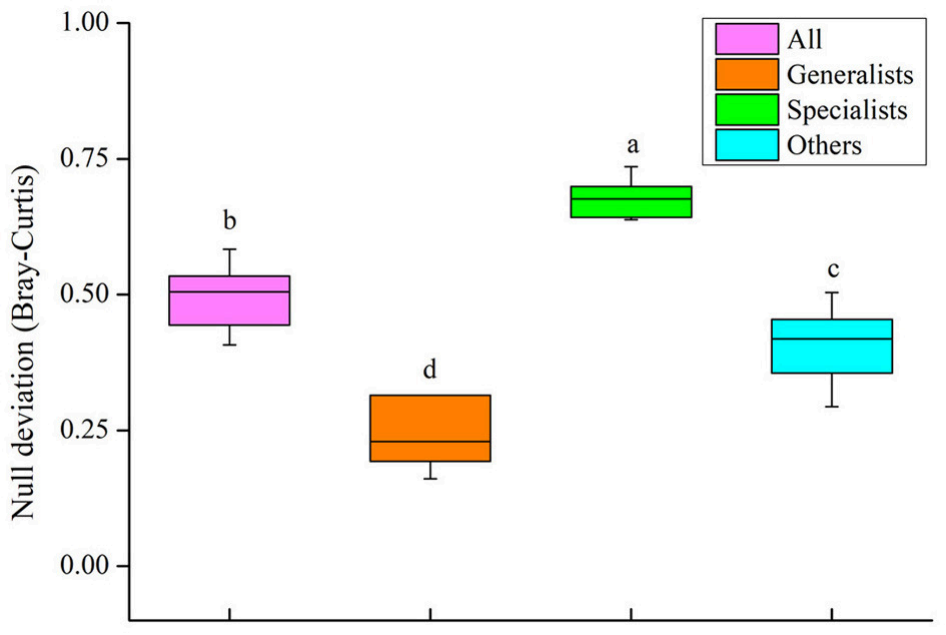
Plots showing the null deviation of soil bacterial community (all) and three subcommunity (generalists, specialists and other taxa). A null deviation close to zero suggests that stochastic processes are more important in structuring the community, whereas larger positive or negative null deviations suggest that deterministic processes are more important.

The relative contribution of both processes structuring the community of the four groups (all, generalists and specialists and other taxa) was tested by the null deviation approach (Figure 9). The results suggested that there were significant differences among the positive β-null deviation values of the four groups (*P* < 0.05, Figure 9). The null deviation values of the entire bacterial community and other taxa were between those of generalists and specialists. Specialists significantly deviated from the stochastic assembly model (relative null deviation = 0.64–0.74) more than generalists (relative null deviation = 0.16–0.65) (*P* < 0.05, Figure 9). When compared with specialists, the results suggested that the stochastic process could played a more significant role in the community assembly of generalists, which was consistent with the results of VPA and the partial Mantel test.

## Discussion

With the application of molecular methods in microbial ecology, the mechanisms explaining elevational patterns of soil microbial diversity and community composition have frequently been published (Shen et al., 2013; Singh et al., 2014; Wang et al., 2015; Yao et al., 2017a). However, the mechanisms driving the elevational distribution patterns of microbial taxa are still not well-understood. In this study, we described the elevational distribution pattern and community assembly of entire bacterial communities and three subcommunities with different niche breadths along the northern slope of Mount Wutai.

### Soil Bacterial Communities Differ Among Elevational Gradients

Our results showed that the dominant phyla were identical among elevational gradients (Figure 2), but that there were significant differences in the taxonomic composition and structure of soil bacterial communities (Figures 2, 4 and Table S4), which was consistent with previous studies of the Changbai Mountain (Shen et al., 2015a; Zhou et al., 2017). In a study by Zhou et al. (2017), elevation showed the strongest total effect on community dissimilarity, followed by competition, soil pH and spatial distance. Shen et al. (2013) found that the bacterial communities differed dramatically along elevations (vegetation types), and the community composition was significantly correlated with soil pH, C/N, moisture or total organic carbon, respectively. Changes in the bacterial community composition along with elevation have previously been attributed to habitat filtering because of lower temperatures toward higher elevations (Wang et al., 2012) or variations in soil pH, moisture and total organic carbon (Shen et al., 2015b). Elevation modifies the local soil physical environment in the rhizosphere (Wallenstein et al., 2007; Djukic et al., 2010) and determines the quantity and quality of litter substrates supply, which may affect the rate of soil organic matter decomposition and indirectly alter the composition of soil bacterial communities (King et al., 2012). In addition to the elevational gradient, we detected shifts in many environmental variables that may influence the bacterial communities including habitat variables, such as pH, ST, TN, TC, and SWC. Our results revealed that elevation, SWC and pH were the most important environmental variables regulating bacterial communities. A recent study conducted in an alpine grassland in the Nyainqentanglha Mountains on the Tibetan Plateau also reported that soil pH had major impacts on bacterial communities (Yuan et al., 2014). Zhang et al. (2013) found that soil moisture, C/N ratio and pH were important determinants of the microbial community structure in Beilu River (on the Tibetan Plateau) permafrost soils with different vegetation types. Soil pH may exert selective pressures on soil bacterial communities by influencing nutrient availability and the rate at which litter can be decomposed. SWC was also an important factor that affected bacterial communities in our study. This was expected given the key effects of moisture on vegetation growth, soil respiration and soil organic carbon content (Wang et al., 2008; Geng et al., 2012), which affect bacterial community composition, especially carbon and nitrogen cycling bacteria (Zheng et al., 2012; Zhang et al., 2014).

Our results also showed that the dominant phyla were identical among elevational gradients, but that the relative abundance of six predominant bacterial phyla (*Acidobacteria, Actinobacteria, Nitrospirae, Bacteroidetes, Firmicutes*, and *Latescibacteria*) were significantly different among elevational gradients (Figure S2). In particular, the relative abundance of *Proteobacteria*, which generally comprises copiotrophic taxa (Francioli et al., 2016; Yao et al., 2017a), did not change across elevational gradients. In contrast, *Acidobacteria*, as the well-defined oligotrophic bacterial phylum (Fierer et al., 2012; Pan et al., 2014; Li et al., 2016), had the greatest relative abundance in low elevational gradient. This may have been because the soil of the dark-coniferous forests at low elevations was relatively nutrient-poor compared to the other two elevational gradients.

### Biogeographic Patterns and Compositions Differ Between Habitat Generalists and Specialists

WHabitat specialization is an important trait that could affect the roles of neutral and niche processes in community assemblies (Pandit et al., 2009). The niche breadth approach (Logares et al., 2013) can identify different levels of habitat specialization of species. Our study showed that bacterial community composition had large heterogeneity between habitat generalists and specialists (Figure 4, Table S4), and generalists exhibited a biogeographic pattern different from that of specialists (Figure 9). More habitat specialists than generalists were found, which agreed with previous observations (Romanuk and Kolasa, 2005; Pandit et al., 2009). Importantly, the phyla of habitat generalists are common in the metacommunity, while those of habitat specialists are rare. Interestingly, many specialist OTUs could not be classified by Silva (Figure 4, Table S4), which suggests many uncultured terrestrial microbes may fulfill these specialist roles *in situ*. When compared to the number of OTUs, the abundance of the most common phyla (*Proteobacteria, Acidobacteria, Actinobacteria* and so on) among generalists was greater than that of specialists (Figure 4, Table S4). Previous studies indicated that *Proteobacteria* and *Actinobacteria* were dominant in soil communities (Glöckner et al., 2000; Janssen, 2006), phylogenetically diverse and widely distributed in water and soil (Freitas et al., 2012; Kindaichi et al., 2016). These organisms have strong adaptability to habitats, and play a role in the degradation of various organic compounds (Zhou et al., 2017). *Acidobacteria* were characterized by slow growing oligotrophy, which could also be an advantage to the ubiquity of microorganisms that are more resistant to nutrient resource changes within their habitat and are thus able to sustain a viable population in heterogeneous environments (Hartman et al., 2008). Moreover, 12 rare phyla only contained in habitat specialists (Figure 4, Table S4). Distinct characteristics in the composition of generalists and specialists might partly explain why they exhibited different elevational diversity (including alpha- and beta- diversity) patterns (Table 2, Figure 5).

Within the generalists subcommunity in the present study, *Proteobacteria, Acidobacteria, Actinobacteria*, and *Chloroflexi* were diverse and abundant bacterial groups. The most abundant bacteria can disperse readily as there are many more individuals that can potentially be involved in a dispersal event (Liu et al., 2015). Specialists are defined by their affiliation with specific habitats (i.e., endemics) or dependence on specific resources, and are more sensitive to environmental variations (Kneitel, 2018). We assumed that the lowest abundance and diversity of specialists in high elevation gradients was because of the harsh environmental conditions (including high solar radiation, large daily temperature fluctuations, high wind exposure, etc.). Several studies (Devictor et al., 2008; Clavel et al., 2011) also revealed that specialists had higher fitness under certain environmental conditions, and that generalists had consistent levels of fitness across a gradient of conditions, consequently, the distribution of specialists varies more across habitats than that of generalists.

### Assembly Mechanisms Differ Between Generalists and Specialists

Our results revealed that the biogeography of the entire bacterial community was significantly shaped by both deterministic processes and spatial factors (Figures 7A, 8A), which was consistent with the results of previous studies (Chase, 2010; Chase and Myers, 2011). Elevation, pH and SWC were found to be important environmental factors regulating the composition of the soil bacterial community (Figure 6A). Therefore, our results suggest that both deterministic (biotic and abiotic environmental filtering) and stochastic (spatial factors) processes drive the soil bacterial communities across elevational gradients on Mount Wutai. More importantly, our results showed that habitat specialists and generalists yielded distinct distribution patterns, and that the importance of environmental variables and spatial factors for bacterial community assembly differed between habitat generalists and specialists (Figures 7–9). The Mantel and partial Mantel test revealed that both environmental variables and spatial factors played significant roles in community assembly of habitat generalists, while only environmental variables influenced shaping of the composition of specialists (Figures 8B,C). VPA showed that abiotic environmental variables explained the greatest proportion of the variation in habitat generalists and specialists (Figures 7B,C). The results of the null deviation approach also corroborated that deterministic processes more strongly explained the assembly of both subcommunities (generalists and specialists) than stochastic processes (Figure 9). This is consistent with niche being an important aspect of species coexistence (Zhou et al., 2017). The effects of stochastic processes (spatial factors) on generalists were significant (Figure 8B), which is consistent with the finding previous studies (Logares et al., 2013; Liao et al., 2016). In contrast to specialists, generalists were more affected by spatial variables (Figures 7, 9), and thus easily followed the random migration from a regional source pool of equivalently fit species. This showed that generalists have a wide habitat tolerance, good exploitation ability, and high functional plasticity (Szekely et al., 2013) and would therefore be less prone to extinction (Székely and Langenheder, 2014).

Generalists are typically not believed to be affected by deterministic processes according to the neutral community model (Logares et al., 2013; Liao et al., 2016). However, in our study, we found that generalists were affected by both spatial factors and environmental factors, and that environmental factors had a greater impact (Figure 7B). A previous study of aquatic microbial communities in rock pools located on an island close to the Swedish Baltic Sea Coast (Székely and Langenheder, 2014) showed that deterministic processes were strongest for habitat generalists. However, studies by Pandit et al. (2009) and Liao et al. (2016) suggested that habitat specialists were only governed by niche processes, whereas habitat generalists were strongly driven by neutral processes. In the present study, spatial factors played a weaker role in the assembly of generalists (Figure 7B), which might have been because of the close distance between sampling points. If so, the relative importance of spatial distance may be reduced relative to other large-scale biogeographical patterns. Importantly, our results showed that only deterministic processes governed the community assembly of specialists (Figure 8C), which was consistent with results of the previous studies that showed habitat specialists could be primarily affected by environmental variables (Desmedt et al., 2014; Liao et al., 2016). These results indicate that specialists might have strict requirements for environmental conditions and that their existence would largely depend on these specific or combined environmental factors. This indicates that specialists might struggle hard to survive under harsh conditions during dispersal and other stochastic processes, and could undergo extinction if drastic abiotic or biotic environmental disturbances occur (Székely and Langenheder, 2014), suggesting that ecological niches and functions of habitat generalists and specialists were not identical.

Elevation, TN and pH were shown to be the main environmental factors determining the habitat generalists, while pH, ${\text{NO}}_{3}^{-}$N, and SWC were significantly related to variations in specialists (Figures 6B,C). Drastic changes in these variables might strongly alter the abundance and composition of generalists and specialists. As the common significant factors for both groups, pH was frequently shown to be an important stressor influencing bacterial composition (Shen et al., 2013; Zhou et al., 2017). SWC and elevation were also found to be significant predictors for bacterial communities, which is in accordance with the results observed in other soil environments (Wang et al., 2008; Geng et al., 2012; Xu et al., 2014). The differences in the environmental factors that significantly affected generalists and specialists likely reflected their different abilities to adapt to the environment.

Under different mechanisms governing their community assembly, specialists, and generalists exhibited disparate biogeographical patterns, diversity and taxonomic compositions. Thus, the different mechanisms governed the community assembly of bacterial taxa with differences in habitat specialization, which might explain why a mixture of deterministic and stochastic processes played a role in the bacterial community assembly (Liao et al., 2016). Furthermore, we deduced that the processes that are most important to the entire community assembly depend on the degree of habitat specialization (Pandit et al., 2009). At the same time, habitat specialization was strongly correlated with different estimates of regional abundance, occurrence and local abundance (Rabinowitz, 1986). Our results demonstrated that the overall amount of variation that could be explained in the entire community tended to be higher for habitat generalists compared with habitat specialists (Figures 7, 8), which indirectly revealed that biogeographical patterns of specialists are more difficult to detect and predict. The variation of specialists explained by both the spatial and environmental factors is relatively low because about 93.3% of the variation is unexplained (Figure 7C). The large proportion of unexplained variation might be caused by additional unmeasured but important environmental factors, temporal factors (Logares et al., 2013) or methodological issues (Dini-Andreote et al., 2015). Another plausible explanation is that biological factors, such as biotic competition, could promote the assembly of bacterial communities (Stegen et al., 2013). Interestingly, our results confirmed that null deviation values of the bacterial subcommunities were positive (Figure 9), which indicated that competition interaction was also a crucial factor in the assembly of soil bacterial communities (Chase, 2010; Chase and Myers, 2011). Goberna et al. (2014) found that competition interaction was more important than abiotic filtration under high resource availability, while abiotic filtration played a more significant role during periods of high environmental stress. In this subalpine mountain study area, the dense covering of litter on the surface of the soil forms an unventilated environment that, when combined with the low soil temperature, is more conducive to the accumulation of soil nutrients (Margesin et al., 2009). Therefore, the relative contributions of biotic interactions cannot be neglected if we are conduct a more systematic investigation of the assembly mechanism of bacterial communities in the future.

## Conclusions

Elucidation of the assembly mechanism of the soil bacterial community is still one of the core issues in microbial ecology. Our study provides important insights for explaining bacterial community patterns in subalpine mountain ecosystems based on niche breadth traits. This study revealed that the compositions of soil bacterial communities among elevational gradients were significantly different, and that both deterministic and stochastic processes played a significant role in the assembly of entire bacterial communities and other taxa. More importantly, the distinct biogeographical patterns of habitat specialists and generalists were underpinned by different mechanisms of community assembly. Deterministic and stochastic processes seemed to determine the assembly and biogeography of habitat generalists, whereas only deterministic processes strongly influenced the distribution of specialists. Our study provides deeper understanding into whether the relative importance of local environmental (selective) vs. spatial (neutral) processes differ for the habitat specialists and generalists. To comprehensively understand the mechanism of bacterial community assembly, it is suggested that in future experiments bacteria be distinguished by the traits of their taxa (e.g., habitat specialization, richness, metabolism, body size, dispersal mode, abundance), and that potential assembly mechanisms among those bacterial taxa across various space and time scales be disentangled.

## Author Contributions

BC and ZL designed the study. PZ, ZL, and JL collected soil samples. ZL, PZ, CL, and BC performed the experiments. TJ and CL provided the plant data. ZL and PZ performed statistical analyses and prepared the draft of the manuscript. BC advised on the Figures and Tables. All the authors revised the manuscript and approved the final version.

### Conflict of Interest Statement

The authors declare that the research was conducted in the absence of any commercial or financial relationships that could be construed as a potential conflict of interest.
