# Supplementary material for: Biogeographic Patterns and Assembly Mechanisms of Bacterial Communities Differ Between Habitat Generalists and Specialists Across Elevational Gradients
Source: Front Microbiol. 2019 Feb 11;10:169. doi: 10.3389/fmicb.2019.00169 (PMC6378303; doi:10.3389/fmicb.2019.00169)
Supplement: Supplementary file 1 [file Table_1.DOC]

**SUPPLEMENTAL MATERIAL**

**Table S1** Brief description of the sampling sites along the northern slope of Mount Wutai.

| Elevational gradient | Site | Elevation (m) | Position | Vegetation type | Dominant species in floor [vegetation](../../../../C:%5CUsers%5Clzm%5CAppData%5CLocal%5CYoudao%5CDict%5CApplication%5C7.5.0.0%5Cresultui%5Cdict%5C%3Fkeyword=vegetation) |
| --- | --- | --- | --- | --- | --- |
| HEG (2900-3055m) | HEG1 | 3050.97 | 113°34'5.927″E 39°4'48.618″N | Alpine meadow | *Kobresia bellardii, Kobresia pygmaea, Thalictrum petaloideum* |
|  | HEG2 | 3031.40 | 113°34'08.708″E 39°4'46.223″N | Alpine meadow | *Kobresia bellardii, Carex spp., Kobresia pygmaea* |
|  | HEG3 | 3019.37 | 113°34'5.464″E 39°4'44.060″N | Alpine meadow | *Kobresia pygmaea, Kobresia bellardii* |
|  | HEG4 | 2950.15 | 113°34'9.403″E 39°4'40.893″N | Subalpine meadow | *Kobresia bellardii, Carex spp., Potentilla spp.* |
|  | HEG5 | 2900.00 | 113°34'3.301″E 39°4'36.722″N | Subalpine meadow | *Carex spp., Kobresia bellard ii* |
| MEG (2400-2800m) | MEG1 | 2728.48 | 113°34'10.485″E 39°4'30.696″N | Subalpine meadow | *Potentilla fruticose, Festuca rubra, Poa annua* |
|  | MEG2 | 2707.02 | 113°34'9.635″E 39°4'26.370″N | Subalpine scrub meadow | *Caragana jubata, Carex spp., Potentilla spp.* |
|  | MEG3 | 2657.01 | 113°34'11.644″E 39°4'23.744″N | Subalpine scrub meadow | *Caragana jubata, Festuca rubra, Poa annua* |
|  | MEG4 | 2581.02 | 113°34'11.953″E 39°4'17.213″N | Subalpine scrub meadow | *Caragana jubata, Festuca rubra, Poa annua* |
| LEG (2000-2300m) | LEG1 | 2295.11 | 113°34'34.586″E 39°3'39.249″N | coniferous forest | *Larix principis-rupprechtii, Carex spp., Thalictrum petaloideum* |
|  | LEG2 | 2221.07 | 113°34'27.479″E 39°3'24.109″N | coniferous forest | *Larix principis-rupprechtii, Carex spp., Polygonum bistorta* |
|  | LEG3 | 2172.08 | 113°34'25.625″E 39°3'14.685″N | coniferous forest | *Larix principis-rupprechtii, Picea, Carex spp., Polygonum bistorta* |
|  | LEG4 | 2085.01 | 113°34'20.064″E 39°2'58.617″N | broad-leaved mixed forest | *Pinus tabulaeformis, Populus davidiana, Carex spp.* |

**Table S2** Environmental variables data of the soil samples from thirteen sites.

| Site | pH | ST(°C) | SM (%) | TN (%) | TC (%) | SOC(g/kg) | C/N ratio | NH_4_^+^-N  (g/kg) | NO_3_^-^-N  (g/kg) | NO_2_^-^-N  (g/kg) | EC(us/cm) | Plant richness |  |
| --- | --- | --- | --- | --- | --- | --- | --- | --- | --- | --- | --- | --- | --- |
| HEG1 | 5.92 | 12.20 | 32.10 | 0.53 | 5.93 | 41.70 | 11.10 | 43.56 | 4.47 | 1.47 | 88.33 | 0.83 | |
| HEG2 | 5.73 | 11.30 | 33.30 | 0.54 | 5.87 | 40.85 | 10.97 | 43.60 | 4.73 | 1.49 | 84.33 | 0.75 | |
| HEG3 | 5.88 | 11.83 | 32.30 | 0.52 | 5.94 | 41.66 | 11.41 | 42.75 | 4.01 | 1.50 | 161.00 | 1.10 | |
| HEG4 | 5.85 | 13.17 | 41.10 | 0.47 | 4.82 | 44.58 | 10.12 | 54.64 | 4.33 | 1.51 | 163.00 | 1.00 | |
| HEG5 | 5.88 | 13.47 | 48.87 | 0.53 | 5.70 | 39.62 | 10.69 | 55.95 | 4.47 | 1.40 | 154.67 | 1.22 | |
| MEG1 | 5.86 | 14.03 | 38.27 | 0.49 | 7.22 | 31.82 | 15.87 | 70.27 | 5.13 | 1.73 | 116.00 | 0.97 | |
| MEG2 | 5.80 | 15.00 | 35.50 | 0.64 | 7.75 | 41.65 | 12.11 | 75.95 | 3.40 | 1.27 | 97.00 | 1.95 | |
| MEG3 | 5.96 | 15.90 | 53.20 | 0.68 | 8.78 | 41.29 | 13.51 | 65.53 | 6.33 | 1.50 | 91.00 | 2.06 | |
| MEG4 | 5.84 | 15.15 | 43.47 | 0.70 | 8.52 | 41.03 | 12.14 | 72.22 | 6.23 | 1.37 | 86.00 | 2.26 | |
| LEG1 | 5.96 | 16.50 | 38.33 | 0.54 | 6.51 | 37.82 | 11.99 | 43.01 | 7.93 | 1.47 | 115.33 | 2.01 | |
| LEG2 | 5.84 | 17.00 | 35.97 | 0.54 | 6.56 | 45.05 | 12.04 | 66.44 | 5.53 | 1.47 | 138.33 | 2.11 | |
| LEG3 | 6.61 | 19.10 | 14.63 | 0.37 | 5.56 | 38.73 | 15.10 | 44.39 | 6.53 | 1.73 | 62.33 | 1.67 | |
| LEG4 | 6.89 | 19.87 | 13.35 | 0.35 | 5.41 | 32.42 | 15.34 | 45.34 | 6.24 | 1.27 | 71.67 | 1.00 | |

**Table S3** ANOSIM statistic of bacterial communities among three elevational gradients.

| Vegetation types | All | |  | Generalists | | |  | Specialists | | | Other taxa | | |
| --- | --- | --- | --- | --- | --- | --- | --- | --- | --- | --- | --- | --- | --- |
|  | r | *P* |  | r | *P* |  | | r | *P* |  | | r | *P* |
| HEG + MEG | **0.3563** | **0.011** |  | 0.06875 | 0.294 |  | | **0.3250** | **0.008** |  | | **0.4250** | **0.007** |
| HEG + LEG | **0.8188** | **0.010** |  | **0.6250** | **0.007** |  | | **0.5375** | **0.014** |  | | **0.9062** | **0.009** |
| MEG + LEG | 0.4375 | 0.061 |  | **0.5000** | **0.026** |  | | 0.1250 | 0.279 |  | | 0.4688 | 0.059 |
| HEG + MEG + LEG | **0.5244** | **0.001** |  | **0.3994** | **0.002** |  | | **0.3425** | **0.004** |  | | **0.5747** | **0.001** |

**Notes:** Bold means significant correlation*（P＜0.05)*.

**Table S4** The OTU compositions of all taxa, specialists, generalists and other taxa within each phylum.

| Taxonomy | All taxa | |  | Generalists | |  | Specialists | |  | Other taxa | |
| --- | --- | --- | --- | --- | --- | --- | --- | --- | --- | --- | --- |
|  | No. of OTU | Total p_i_ (%) |  | No. of OTU | Total p_i_ (%) |  | No. of OTU | Total p_i_ (%) |  | No. of OTU | Total p_i_ (%) |
| *Proteobacteria* | 682 | 27.01 |  | 109 | 14.14 |  | 50 | 0.22 |  | 523 | 12.64 |
| *Acidobacteria* | 391 | 21.74 |  | 46 | 7.77 |  | 29 | 0.18 |  | 316 | 13.80 |
| *Actinobacteria* | 331 | 17.19 |  | 27 | 2.46 |  | 15 | 0.09 |  | 289 | 14.64 |
| *Chloroflexi* | 352 | 14.53 |  | 34 | 5.69 |  | 41 | 0.29 |  | 277 | 8.55 |
| *Nitrospirae* | 39 | 4.46 |  | 9 | 2.59 |  | 2 | 5.22×10^-3^ |  | 28 | 1.87 |
| *Gemmatimonadetes* | 83 | 2.91 |  | 16 | 2.20 |  | 5 | 0.02 |  | 62 | 0.69 |
| *Bacteroidetes* | 202 | 2.79 |  | 20 | 0.73 |  | 37 | 0.38 |  | 145 | 1.67 |
| *Verrucomicrobia* | 82 | 2.53 |  | 7 | 0.51 |  | 5 | 0.02 |  | 70 | 2.01 |
| *Firmicutes* | 88 | 1.37 |  | 0 | 0.00 |  | 33 | 0.13 |  | 55 | 1.24 |
| *Parcubacteria* | 108 | 1.15 |  | 3 | 0.51 |  | 21 | 0.08 |  | 84 | 0.94 |
| unclassified_k__norank | 63 | 1.01 |  | 2 | 0.07 |  | 24 | 0.68 |  | 37 | 0.26 |
| *Planctomycetes* | 116 | 0.88 |  | 3 | 0.13 |  | 9 | 0.03 |  | 104 | 0.71 |
| *Latescibacteria* | 36 | 0.67 |  | 2 | 0.05 |  | 1 | 2.79×10^-3^ |  | 33 | 0.61 |
| *Saccharibacteria* | 73 | 0.57 |  | 0 | 0.00 |  | 8 | 0.11 |  | 65 | 0.46 |
| *Cyanobacteria* | 36 | 0.36 |  | 0 | 0.00 |  | 7 | 0.03 |  | 29 | 0.33 |
| *Tectomicrobia* | 4 | 0.10 |  | 0 | 0.00 |  | 0 | 0.00 |  | 4 | 0.10 |
| *WS6* | 13 | 0.09 |  | 0 | 0.00 |  | 2 | 4.53×10^-3^ |  | 11 | 0.09 |
| *Chlorobi* | 16 | 0.08 |  | 0 | 0.00 |  | 1 | 6.27×10^-3^ |  | 15 | 0.08 |
| *Armatimonadetes* | 14 | 0.08 |  | 0 | 0.00 |  | 1 | 2.09×10^-3^ |  | 13 | 0.08 |
| *WS2* | 4 | 0.07 |  | 1 | 0.02 |  | 0 | 0.00 |  | 3 | 0.05 |
| *Ignavibacteriae* | 6 | 0.07 |  | 0 | 0.00 |  | 1 | 6.27×10^-3^ |  | 5 | 0.06 |
| *Elusimicrobia* | 12 | 0.06 |  | 1 | 0.01 |  | 0 | 0.00 |  | 11 | 0.049 |
| *Chlamydiae* | 13 | 0.06 |  | 0 | 0.00 |  | 4 | 0.01 |  | 9 | 0.046 |
| *Spirochaetae* | 5 | 0.04 |  | 0 | 0.00 |  | 3 | 0.02 |  | 2 | 0.02 |
| *Fibrobacteres* | 5 | 0.03 |  | 0 | 0.00 |  | 2 | 0.02 |  | 3 | 0.01 |
| *Fusobacteria* | 2 | 0.03 |  | 0 | 0.00 |  | 0 | 0.00 |  | 2 | 0.03 |
| *TM6__Dependentiae_* | 8 | 0.03 |  | 0 | 0.00 |  | 2 | 4.53×10^-3^ |  | 6 | 0.02 |
| *BRC1* | 4 | 0.02 |  | 0 | 0.00 |  | 0 | 0.00 |  | 4 | 0.02 |
| *Omnitrophica* | 3 | 0.02 |  | 0 | 0.00 |  | 0 | 0.00 |  | 3 | 0.02 |
| *Gracilibacteria* | 4 | 0.01 |  | 0 | 0.00 |  | 0 | 0.00 |  | 4 | 0.01 |
| *Nitrospinae* | 2 | 0.01 |  | 0 | 0.00 |  | 0 | 0.00 |  | 2 | 0.01 |
| *GAL15* | 1 | 0.01 |  | 0 | 0.00 |  | 0 | 0.00 |  | 1 | 4.53×10^-3^ |
| *BJ-169* | 1 | 4.53×10^-3^ |  | 0 | 0.00 |  | 0 | 0.00 |  | 1 | 4.53×10^-3^ |
| *WWE3* | 1 | 4.53×10^-3^ |  | 0 | 0.00 |  | 0 | 0.00 |  | 1 | 3.83×10^-3^ |
| *Candidatus_Berkelbacteria* | 1 | 3.83×10^-3^ |  | 0 | 0.00 |  | 0 | 0.00 |  | 1 | 3.83×10^-3^ |
| *FCPU426* | 1 | 3.83×10^-3^ |  | 0 | 0.00 |  | 0 | 0.00 |  | 1 | 3.83×10^-3^ |
| *Tenericutes* | 1 | 3.83×10^-3^ |  | 0 | 0.00 |  | 1 | 3.83×10^-3^ |  | 0 | 0.00 |
| *CPR2* | 1 | 3.48×10^-3^ |  | 0 | 0.00 |  | 1 | 3.48×10^-3^ |  | 0 | 0.00 |
| norank | 1 | 2.79×10^-3^ |  | 0 | 0.00 |  | 0 | 0.00 |  | 1 | 0.00279 |
| *Microgenomates* | 1 | 2.44×10^-3^ |  | 0 | 0.00 |  | 0 | 0.00 |  | 1 | 0.00244 |
| *Cloacimonetes* | 1 | 2.44×10^-3^ |  | 0 | 0.00 |  | 1 | 2.44×10^-3^ |  | 0 | 0.00 |
| *Peregrinibacteria* | 1 | 2.09×10^-3^ |  | 0 | 0.00 |  | 1 | 2.09×10^-3^ |  | 0 | 0.00 |

**Notes:** *p_i_ represents the mean relative abundance


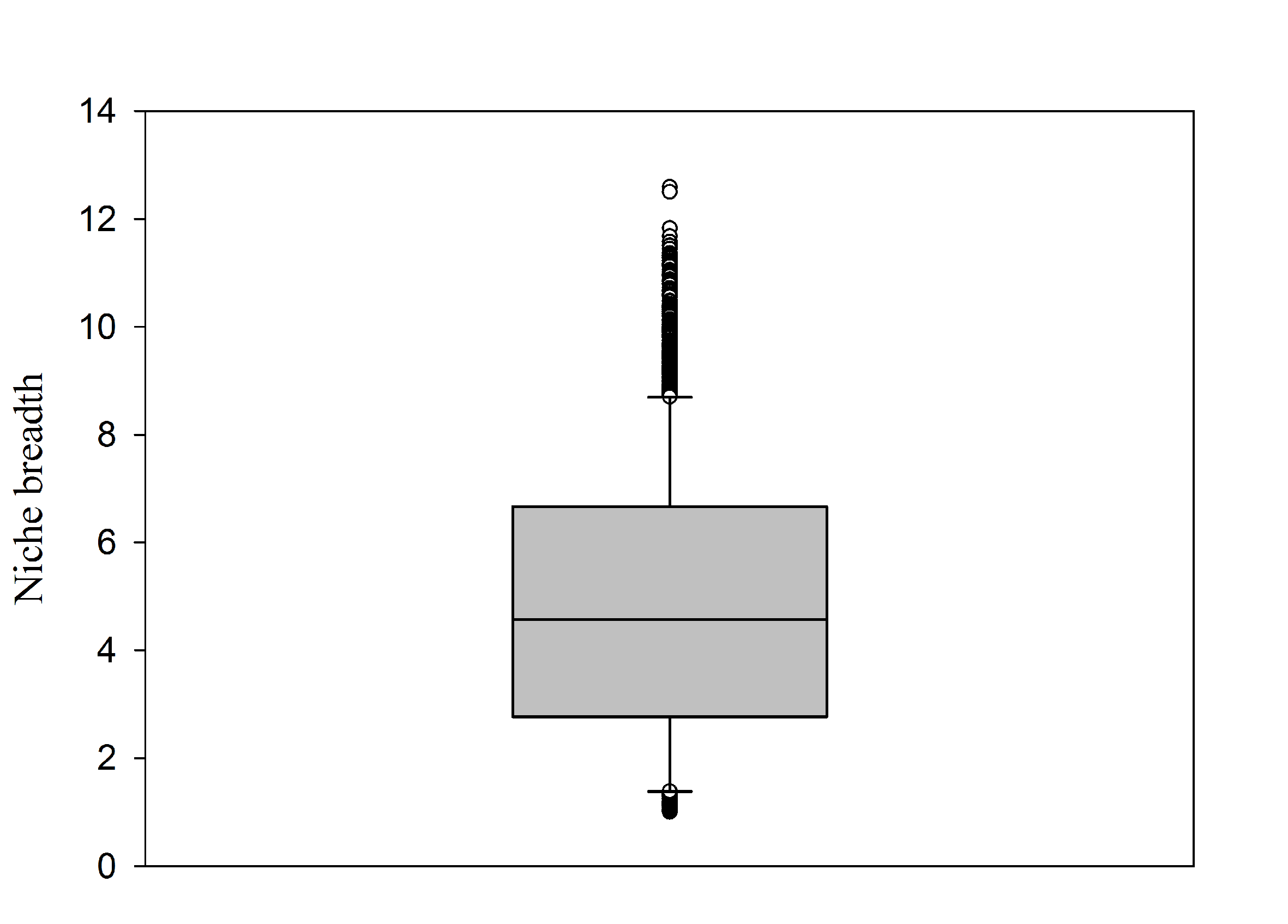


**Fig S1** Distribution of niche breadth (B) values of OTUs. This B-value >8.7 criterion was chosen as generalists because this value lies within the outlier area of the B-value distribution. Meanwhile, OTUs with B-value <1.5 were regarded as specialists.


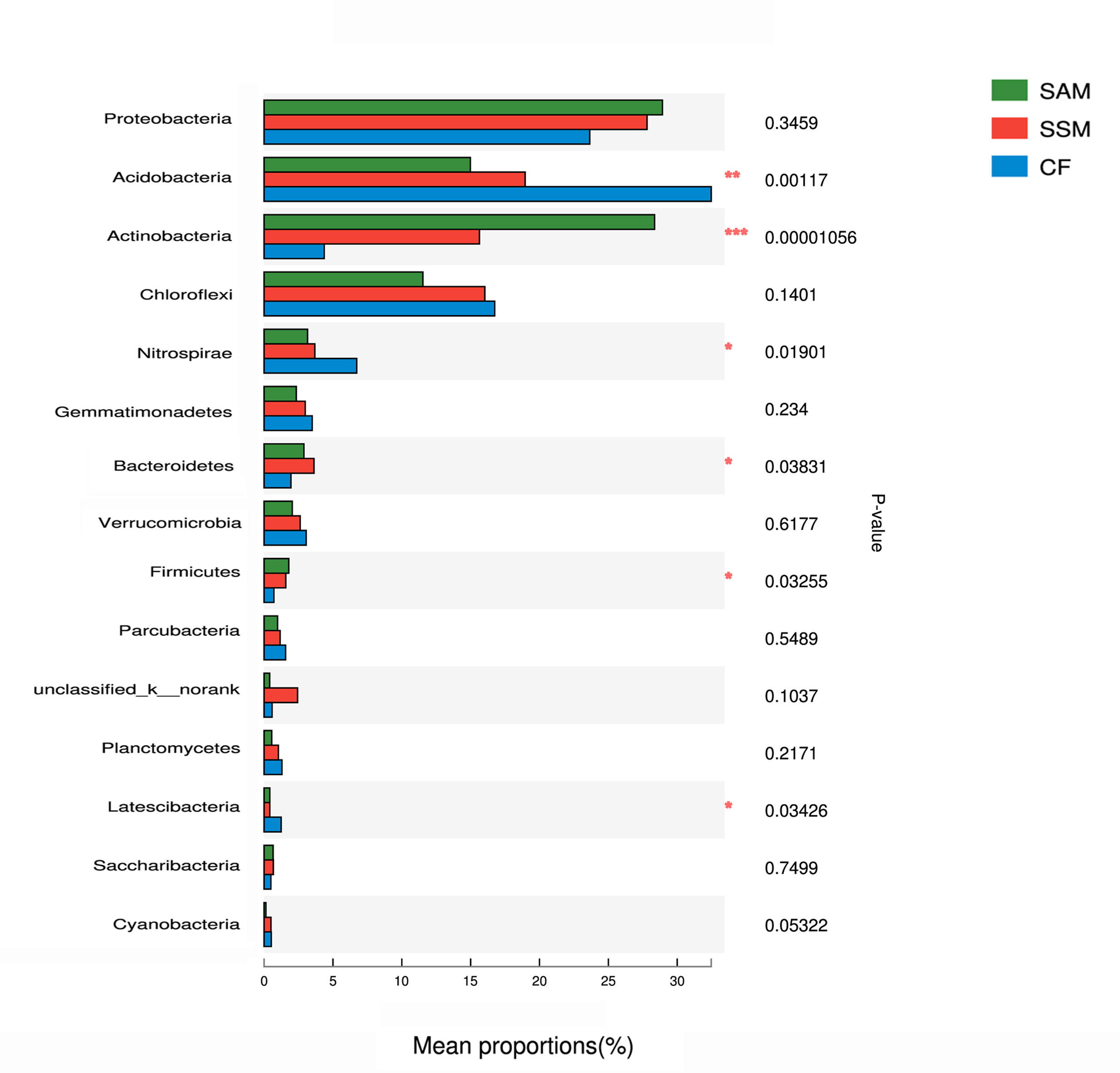


**Fig S2** Differences in the relative abundance of the dominant bacterial phyla among three elevational gradients.

*** indicate *P* < 0.001; ** indicate *P* <0 .01; * indicate *P* <0 .05.
